# 

**Published:** 1903-05

**Authors:** 


***NOTICE.— This Journal and the International Journal of Surgery one year for $1.00 cash, to new subscribers. No out-of-town checks accepted unless 10c. is added for cost of cashing. Strictly to new subscribers.***

---

**This Journal and Atlanta Semi-weekly Journal one year for $1.25 cash in advance. No checks. Strictly to new subscribers.**

